# Retraction for Hiramatsu et al., “Melanin Produced by *Bordetella parapertussis* Confers a Survival Advantage to the Bacterium during Host Infection”

**DOI:** 10.1128/msphere.01072-24

**Published:** 2025-02-05

**Authors:** Yukihiro Hiramatsu, Takashi Nishida, Dendi Krisna Nugraha, Fuminori Sugihara, Yasuhiko Horiguchi

## RETRACTION

Volume 6, no. 5, e00819-21, 2021, https://doi.org/10.1128/msphere.00819-21. An investigation conducted by the senior author’s laboratory has concluded that the data shown in most of the figures are the results of fabricated experiments or extensive falsification. An official investigation is ongoing at Osaka University, and certain results will require further verification by re-experimentation. Therefore, we are retracting the article and regret any inconvenience this may have caused.

